# Photobiomodulation to minimize talquetamab-associated dysgeusia and xerostomia in multiple myeloma: a brief report of four cases

**DOI:** 10.1007/s00520-026-11133-8

**Published:** 2026-08-22

**Authors:** Mariana Henriques Ferreira, Fernanda de Paula Eduardo, Luiza Deitos Menti, Isabela Vieira Nasser de Morais, Nelson Hamerschlak, Mariana Nassif Kerbauy, Gabriela Rocco de Sá, Letícia Mello Bezinelli

**Affiliations:** 1 Hospital Israelita Albert Einstein, São Paulo, Brazil; 2 Faculdade Israelita de Ciências da Saúde Albert Einstein, Hospital Israelita Albert Einstein, São Paulo, Brazil

**Keywords:** Photobiomodulation, Talquetamab, Dysgeusia, Xerostomia, Multiple myeloma, Supportive care

## Abstract

**Background:**

Talquetamab, a GPRC5D-targeting bispecific antibody, produces high response rates in relapsed/refractory multiple myeloma (RRMM) but is frequently associated with oral adverse effects, most commonly dysgeusia and xerostomia, which can impair nutrition and quality of life. No evidence-based supportive-care intervention has been established for these effects.

**Methods:**

We describe four adults with RRMM who received photobiomodulation (PBM) concomitantly with talquetamab, using a dual-wavelength (660/808 nm) protocol individualized to symptom evolution and patient availability. Dysgeusia and xerostomia were assessed prospectively at each session through structured clinical interview and oral examination, with severity graded for each patient; no formal psychophysical gustatory testing or sialometry was performed. This descriptive, uncontrolled case series was designed to report the feasibility and tolerability of PBM rather than its efficacy.

**Results:**

All four patients developed dysgeusia during talquetamab treatment. Two had grade 1, intermittent hypogeusia; two had grade 2 dysgeusia with complete taste loss (ageusia). No patient developed oral mucositis or clinically significant oral pain. All four patients reported xerostomia, with reduced salivary film and whitish, viscous saliva on examination; salivary flow was not measured quantitatively. All patients also received xylitol-based mouthwash and saliva substitutes from the start of talquetamab as concomitant standard supportive care. PBM was well tolerated, with no PBM-related adverse events, and no patient discontinued talquetamab because of oral toxicity.

**Conclusions:**

This series demonstrates the feasibility and tolerability of PBM delivered alongside talquetamab in four patients. Given the small, uncontrolled sample and concurrent supportive interventions, no conclusion can be drawn regarding the efficacy of PBM for talquetamab-associated dysgeusia or xerostomia. Controlled studies with objective, validated outcome measures are needed.

**Supplementary Information:**

The online version contains supplementary material available at https://doi.org/10.1007/s00520-026-11133-8.

## Introduction

Multiple myeloma (MM) is a hematologic malignancy characterized by clonal proliferation of plasma cells that remains incurable despite recent advances in therapy. Over the past decade, immunotherapies—particularly bispecific antibodies and chimeric antigen receptor (CAR)-T cell therapies targeting B-cell maturation antigen (BCMA) and G protein-coupled receptor, class C, group 5, member D (GPRC5D)—have substantially improved survival outcomes. Talquetamab, a first-in-class GPRC5D-directed bispecific antibody, has demonstrated high response rates in patients with relapsed and/or refractory MM (RRMM), offering a new therapeutic option for heavily pretreated patients [1]. Talquetamab safety profile includes a distinct pattern of oral adverse effects not commonly seen with other myeloma therapies. In pivotal trials, dysgeusia and xerostomia were reported in up to 60–67% of patients, likely related to GPRC5D expression in non-malignant tissues such as the filiform papillae of the tongue and minor salivary glands [2]. These manifestations can impair oral function, nutrition, and quality of life, and may contribute to reduced food intake, weight loss, or treatment discontinuation.

There is currently no evidence-based intervention specifically targeting talquetamab-associated dysgeusia and xerostomia; conventional measures such as saliva substitutes, mouth rinses, and moisturizing sprays offer only partial or temporary relief. Photobiomodulation (PBM) is used in oncology for the prevention and treatment of oral mucositis and treatment-related neuropathic symptoms, with proposed anti-inflammatory, regenerative, and neuromodulatory effects mediated by mitochondrial activation and improved microcirculation [3]. PBM has also been reported to increase salivary flow in xerostomia of other etiologies, such as Sjögren’s syndrome [4]. These mechanisms provide biological plausibility—but not evidence—for a possible role of PBM in talquetamab-associated dysgeusia and xerostomia. This report aims to describe the use of PBM in four patients with RRMM who developed these oral adverse effects during talquetamab treatment.

## Methods

This descriptive case series includes four adult patients (≥ 18 years) with RRMM who received talquetamab between August 2024 and October 2025 at Hospital Israelita Albert Einstein, São Paulo, Brazil, and who received PBM for talquetamab-associated dysgeusia and/or xerostomia. Relevant items of the CARE guidelines for case report/case series reporting were followed where applicable; a completed CARE checklist is provided as supplementary material. The study was approved by the institutional ethics committee.

All consecutive adult patients who received talquetamab at our institution during the study period and developed dysgeusia and/or xerostomia were offered PBM; no eligible patient was excluded from this series.

### Eligibility criteria

Patients were eligible if they were receiving talquetamab as inpatients or outpatients and were clinically able to undergo intraoral examination. Exclusion criteria were pre-existing conditions known to affect taste perception (neurologic disorders, prior head and neck radiotherapy), taste alteration present before talquetamab initiation, and contraindications to PBM such as active premalignant or malignant oral lesions or concomitant use of photosensitizing medications.

### Photobiomodulation protocol

PBM was initiated concomitantly with the first talquetamab administration and continued during hospitalization or scheduled outpatient visits, using a continuous-wave (CW) gallium-aluminum-arsenide (GaAlAs) diode laser (DMC®, São Carlos, Brazil) that emits two wavelengths simultaneously from the same probe: 660 nm, targeting the superficial epithelium and lingual papillae, and 808 nm, targeting deeper neural and anti-inflammatory effects. Because both wavelengths are emitted concurrently from a single probe, every application point received both wavelengths identically, and applications were performed in direct contact, perpendicular to the mucosa.

Each wavelength was delivered at a power output of 100 mW through a probe with a spot area of 0.4 cm^2^ (≈7.1 mm diameter), applied in contact mode. Because the two wavelengths are emitted simultaneously, an irradiation time of 10 s at each point delivered 1 J from the 660 nm diode plus 1 J from the 808 nm diode, for a fixed combined energy of 2 J per point. This corresponds to an irradiance of 0.25 W/cm^2^ per wavelength (0.5 W/cm^2^ combined) and a combined energy density (fluence) of approximately 5 J/cm^2^ per point (2.5 J/cm^2^ per wavelength).

**Fig. 1:**
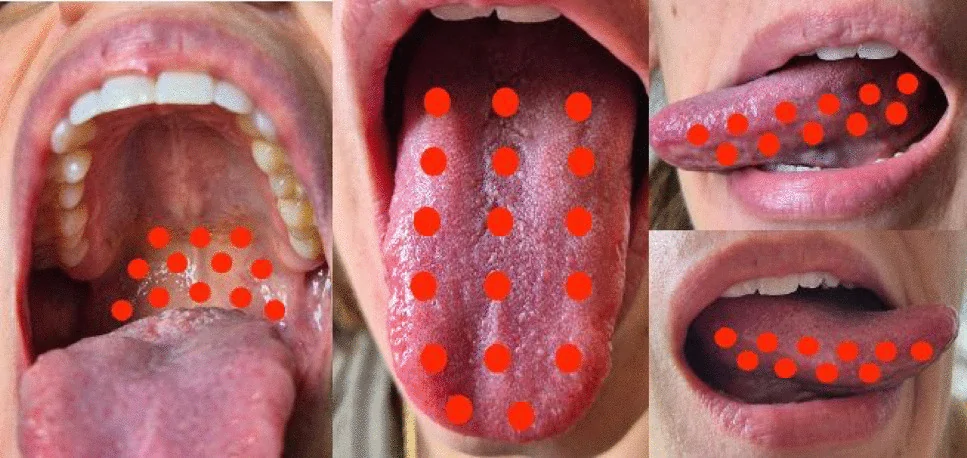
Representative clinical photographs, not from any of the four patients included in this series, used for illustrative purposes only to show the intraoral distribution of photobiomodulation application points (red markers) on the floor of the mouth/soft palate, dorsal tongue, and bilateral lateral tongue. Because the device emits both wavelengths (660 nm and 808 nm) simultaneously from the same probe, every point received an identical, combined application, and markers are therefore not color-coded by wavelength

Points were spaced 1 cm apart across the dorsal tongue (15 points), bilateral lateral tongue (10 points per side), and soft palate (10 points), for a maximum of 45 points per full session (Fig. 1); the number of points treated was adjusted according to individual anatomical variation in tongue size. Sessions were delivered up to five times per week during the first days after each talquetamab administration—coinciding with the step-up dosing schedule and the period of highest risk for oral toxicity—and were subsequently spaced to approximately twice weekly as symptoms stabilized or resolved, according to patient availability and clinical tolerance. The number of PBM sessions per patient and the total cumulative energy delivered therefore varied according to the number of sessions and points treated and are reported individually in Table 1. PBM was performed under aseptic conditions by oral medicine specialists trained and calibrated in the study protocol, using standardized infection-control measures. A complete dosimetry reporting summary is provided in Table 2.

**Table 1:** Individual patient characteristics, PBM protocol, and clinical course

Variable	Patient 1	Patient 2	Patient 3	Patient 4
Age/sex	94/female	54/female	70/female	46/female
Myeloma status (stage, prior lines of therapy)	Relapsed/fifth-line treatment	Refractory/fourth-line treatment	Relapsed/sixth-line treatment	Refractory/fourth-line treatment
Talquetamab schedule and dose	0.8 mg/kg SC, every 15 days (Q2W maintenance)	0.8 mg/kg SC, every 15 days (Q2W maintenance)	0.8 mg/kg SC, every 15 days (Q2W maintenance)	0.8 mg/kg SC, every 15 days (Q2W maintenance)
Duration of talquetamab exposure at data cutoff	24 weeks (active)	16 weeks (discontinued as scheduled)	28 weeks (active)	20 weeks (active)
Baseline oral examination findings	Intact mucosa, normal lingual papillae, adequate salivary film, no erythema/ulceration	Intact mucosa, normal lingual papillae, adequate salivary film, no erythema/ulceration	Intact mucosa, mild coated tongue, adequate salivary film, no erythema	Intact mucosa, normal lingual papillae, adequate salivary film, no erythema/ulceration
Onset of dysgeusia (day) and pattern	Day 8	Day 6	Day 10	Day 7
Dysgeusia grade/severity and duration	Grade 1 (mild hypogeusia); 14 days, intermittent, preserved taste discrimination	Grade 2 (complete taste loss/ageusia); 5 days after drug discontinuation	Grade 1 (mild hypogeusia); 10 days, intermittent periods of ageusia	Grade 2 (complete taste loss from day 6 post-ramp-up); 8 days
Xerostomia onset and grade	Day 5/grade 1	Day 4/grade 2	Day 7/grade 1	Day 5/grade 1
Duration of xerostomia	12 days (transient, managed with oral lubricants)	18 days (transient, resolved after supportive care)	10 days (transient, managed with oral lubricants)	14 days (transient, resolved after supportive care)
PBM start relative to talquetamab	Day 1 (concomitant with 1 st dose)	Day 1 (concomitant with 1 st dose)	Day 1 (concomitant with 1 st dose)	Day 1 (concomitant with 1 st dose)
Total PBM sessions	12	8	14	10
Wavelength(s) applied per point and per session	660 + 808 nm, simultaneous	660 + 808 nm, simultaneous	660 + 808 nm, simultaneous	660 + 808 nm, simultaneous
Energy per point (J)	2	2	2	2
Concomitant supportive measures for xerostomia	Xylitol rinse + oral lubricant	Xylitol rinse + oral lubricant	Xylitol rinse + oral lubricant	Xylitol rinse + oral lubricant
Nutritional impact/weight change	− 0.5 kg	− 1.2 kg	+ 0.2 kg	− 0.8 kg
Treatment interruptions (talquetamab or PBM)	None	None (talquetamab discontinued as scheduled per protocol; no PBM interruption)	None	None
Follow-up after talquetamab discontinuation or data cutoff	12 weeks post-cut off; normal taste and salivary perception maintained	8 weeks post-discontinuation; complete resolution of dysgeusia by day 5 post-treatment	10 weeks post-cut off; sustained oral comfort, no late oral toxicities	8 weeks post-cut off; full recovery of taste perception and baseline salivary comfort

**Table 2:** Standard photobiomodulation dosimetry reporting parameters

Parameter	Value/description
Laser type	Diode, GaAlAs (DMC®, São Carlos, Brazil)
Wavelengths	660 nm (visible red) and 808 nm (near-infrared), emitted simultaneously
Operating mode	Continuous wave (CW)
Output power	100 mW per wavelength (200 mW combined)
Spot size	0.4 cm^2^ (≈7.1 mm diameter)
Irradiance (power density)	0.25 W/cm^2^ per wavelength (0.5 W/cm^2^ combined)
660 nm energy & duration per point	10 s = 1.0 J/point; fluence ≈2.5 J/cm^2^
808 nm energy & duration per point	10 s = 1.0 J/point; fluence ≈2.5 J/cm^2^
Combined energy & fluence per point	2.0 J/point; fluence ≈5 J/cm^2^
Application mode	Direct contact, perpendicular to mucosa
Application sites	Dorsal tongue (15 points), lateral tongue (10 points per side), soft palate (10 points)

The energy parameters used are within the ranges proposed by MASCC/ISOO clinical practice guidelines for photobiomodulation in the supportive care of head and neck cancer treatment-related complications, including dysgeusia and dry mouth [5, 6]; those guidelines were developed for chemoradiation-related toxicity in head and neck cancer, and no guideline currently addresses talquetamab-associated dysgeusia or xerostomia specifically.

### Clinical evaluation

Dysgeusia was assessed prospectively at each PBM session through a structured clinical interview, in which patients reported the presence, quality (metallic, bitter, salty, or absent taste), and severity of taste alterations, together with any impact on food intake. Severity was graded according to the symptomatic and functional descriptors of the Common Terminology Criteria for Adverse Events (CTCAE v5.0): for dysgeusia, grade 1 (altered taste without a change in diet) or grade 2 (altered taste with a change in diet, or loss of taste); for xerostomia, grade 1 (symptomatic, without significant dietary alteration) or grade 2 (moderate symptoms with dietary adaptation or use of oral lubricants). The salivary flow rate component of the CTCAE xerostomia criteria could not be applied, as sialometry was not performed. No formal psychophysical gustatory test (e.g., Burghart taste strips) was used. Xerostomia was further characterized by clinical inspection of salivary film and mucosal moisture. Data were recorded prospectively in standardized case report forms.

On clinical oral examination, no patient showed signs of oral candidiasis, and no patient had documented anemia during the observation period; these findings, however, were based on clinical impression and available records rather than a standardized assessment systematically recorded at every visit.

### Safety assessment

Adverse events potentially related to PBM—such as pain, erythema, or burning sensation—were documented at each session. No topical anesthetics or photosensitizing agents were used.

### Ethics and consent

This study was approved by the Institutional Review Board of Hospital Israelita Albert Einstein (São Paulo, Brazil). Written informed consent for publication of clinical data and images was obtained from all four patients.

## Results

Four adult patients with RRMM receiving talquetamab were included; individual characteristics, PBM protocols, and clinical course are summarized in Table 1.

All four patients developed dysgeusia during talquetamab treatment, with onset between day 6 and day 10 after the first dose (Table 1). Two patients (Patients 1 and 3) had CTCAE grade 1, intermittent hypogeusia with preserved discrimination of basic tastes, lasting 10–14 days; the other two (Patients 2 and 4) had CTCAE grade 2 dysgeusia with complete taste loss (ageusia), lasting 5–8 days. No patient developed oral mucositis or clinically significant oral pain. No patient discontinued talquetamab because of oral adverse effects; one patient (Patient 2) discontinued talquetamab as scheduled per the treatment plan, unrelated to oral toxicity.

All four patients reported xerostomia (CTCAE grade 1 in three patients and grade 2 in one), with onset between day 4 and day 7 and duration of 10–18 days (Table 1). Clinical examination showed reduced salivary film with whitish, viscous saliva in all four patients; because salivary flow was not measured quantitatively, grading relied on symptomatic and functional criteria only, and this reflects a clinical impression rather than a confirmed reduction in salivary output. All four patients received daily xylitol-based mouthwash and saliva substitutes from the first day of talquetamab administration; patients were instructed on their correct use both verbally and in writing, and reported adherence during structured interviews, but adherence was not independently verified.

PBM was delivered without interruption in all four patients, and no PBM-related adverse event was observed.

## Discussion

In this four-patient descriptive series, all patients developed talquetamab-associated dysgeusia, and all developed xerostomia. Because PBM was applied concomitantly with talquetamab from treatment initiation in every patient, without an untreated or historical comparator, and because all patients also received xylitol-based mouthwash and saliva substitutes, this series cannot determine whether PBM attenuated dysgeusia, accelerated recovery, influenced salivary symptoms, or prevented mucositis. The clinical course observed—predominantly intermittent taste alteration with preserved basic taste discrimination on most days, absence of mucositis, and no treatment discontinuation for oral reasons—should be interpreted as a description of this cohort's course, not as evidence of a PBM effect. The temporal association between talquetamab initiation and symptom onset, together with normal baseline oral findings in all four patients (Table 1) and resolution of symptoms after drug discontinuation in one patient, supports a treatment-emergent etiology rather than alternative diagnoses such as primary burning mouth syndrome, which is typically chronic and not temporally linked to a new systemic therapy. Comparable dysgeusia frequencies have been reported elsewhere: [7] observed taste alteration in 96.2% of 26 patients receiving talquetamab; [2] reported taste disturbance in 57–63% of patients. These studies differ from ours in population, PBM use, and outcome assessment, and are cited here only as descriptive context, not as a basis for inferring a treatment effect. [8] reported reversal of talquetamab-associated dysgeusia after PBM in a single case using a different energy density (25 J/cm^2^ per field) than the present series (2 J per point, ≈5 J/cm^2^). Given the differences in design, population size, and protocol between that report and the present series, this single case does not permit any inference about the reproducibility or comparability of PBM's effects across studies.

PBM is proposed to act through photobiological modulation of mitochondrial metabolism, with anti-inflammatory, angiogenic, and regenerative effects that have been hypothesized to support epithelial and neural recovery, and preclinical work has shown taste bud regeneration after chemotherapy-induced injury [9]. These mechanisms may be biologically plausible explanations for a potential effect of PBM on taste and salivary function, but none was measured in the present series—we assessed neither mitochondrial function, neural recovery, taste bud morphology, salivary flow, quality of life, weight, nor treatment adherence. These mechanistic considerations should therefore be regarded as hypotheses for future study rather than as explanations demonstrated by this report.

Xerostomia in this population is likely multifactorial. Proposed contributors include GPRC5D expression in minor salivary glands, concurrent medications, and other patient-specific factors; xylitol-based mouthwash and saliva substitutes were used by all four patients from the start of talquetamab and may independently have influenced oral comfort and food intake. Because salivary flow was not measured quantitatively, we cannot distinguish subjective xerostomia from clinically confirmed hyposalivation, nor attribute any change to PBM rather than to concomitant supportive measures. PBM has been reported to increase salivary flow in xerostomia related to Sjögren’s syndrome [4]; this differs substantially in etiology from talquetamab-associated xerostomia and is noted here only as rationale for future controlled study of PBM across xerostomia etiologies, not as supporting evidence for the present series.

This report has several limitations. The sample is small (*n* = 4) and uncontrolled, without an untreated or historical comparator, precluding causal inference. Dysgeusia and xerostomia were assessed through structured clinical interview and examination with CTCAE-based symptomatic/functional grading, rather than through formal psychophysical gustatory testing or sialometry; in particular, the flow rate component of the CTCAE xerostomia criteria could not be applied. Validating these findings with objective, quantitative instruments is an important direction for future studies. PBM session frequency (up to five times weekly, tapering to approximately twice weekly as symptoms stabilized) varied according to patient availability and clinical course rather than a fixed protocol, and the number of sessions and points treated varied among patients; this heterogeneity in total cumulative exposure limits comparison across patients and with other reports, even though the energy delivered per point (2 J) was fixed. All patients received concurrent xylitol mouthwash and saliva substitutes, and adherence to these measures was based on self-report rather than independently verified; this cannot be separated from any effect attributed to PBM. Although no patient showed clinical signs of oral candidiasis or documented anemia, these parameters were not part of a standardized assessment systematically recorded at every visit.

In this series, PBM sessions were empirically concentrated during the first days after each talquetamab administration—coinciding with the step-up dosing schedule and the period of highest risk for oral toxicity—and then spaced to approximately twice a week as symptoms stabilized. Although this pattern was applied pragmatically rather than tested against a fixed-frequency comparator, it suggests an induction-then-maintenance strategy (intensive PBM during the acute symptomatic window, followed by a reduced maintenance schedule) that could be formally evaluated in future controlled studies; this approach has precedent in other PBM applications, such as chronic oral graft-versus-host disease [10].

As GPRC5D- and other novel-target immunotherapies become more widely used, oral adverse effects such as dysgeusia and xerostomia are emerging as relevant supportive-care concerns, and current management options remain largely palliative [11, 12]. PBM is a non-pharmacologic intervention with an established safety profile in other oncology indications; whether it has a therapeutic role in talquetamab-associated oral adverse effects remains to be determined. The safety and feasibility observed in this series support the design of a prospective, controlled study, which the authors are considering to formally evaluate the efficacy of PBM in this setting.

## Conclusion

This descriptive series shows that PBM can be delivered safely and is well tolerated when administered concomitantly with talquetamab in patients with RRMM. It does not establish that PBM prevents, attenuates, or accelerates recovery from talquetamab-associated dysgeusia or xerostomia. Controlled, prospective studies using validated gustatory and salivary flow measures, and explicitly accounting for concurrent supportive interventions, are needed to determine whether PBM has a therapeutic role in this setting.

## Supplementary Information

Below is the link to the electronic supplementary material.

ESM 1 (DOCX 12.8 KB)

## Data Availability

The datasets generated and/or analyzed during the current study are not publicly available due to patient privacy and institutional restrictions but are available from the corresponding author on reasonable request.
