# Supplementary material for: Photobiomodulation to minimize talquetamab-associated dysgeusia and xerostomia in multiple myeloma: a brief report of four cases
Source: Support Care Cancer. 2026 Aug 22;34(9):888. doi: 10.1007/s00520-026-11133-8 (PMC13498624; doi:10.1007/s00520-026-11133-8)
Supplement: Supplementary file 1 [file 520_2026_11133_MOESM1_ESM.docx]

**CARE Checklist**

*Manuscript: Photobiomodulation to Minimize Talquetamab-Associated Dysgeusia and Xerostomia in Multiple Myeloma: A Brief Report of Four Cases*

*Note: CARE was developed for single-patient case reports. As stated in the Methods, relevant CARE items were applied, where appropriate, to this four-patient descriptive case series; items are reported once per manuscript (not once per patient) except where table-based per-patient reporting is indicated below.*

| **#** | **Topic** | **CARE item** | **Reported in manuscript** |
| --- | --- | --- | --- |
| 1 | Title | The area of focus and "case report"/equivalent descriptor are identified in the title. | Title page. "...A Brief Report of Four Cases" identifies both the focus (PBM for talquetamab-associated dysgeusia/xerostomia) and the report type. |
| 2 | Key words | 2–5 key words that identify diagnoses/interventions. | Keywords, after Abstract: Photobiomodulation; Talquetamab; Dysgeusia; Xerostomia; Multiple Myeloma; Supportive Care. |
| 3a | Abstract – Introduction | What is unique about this case/series and what does it add to the literature? | Abstract, Background. |
| 3b | Abstract – Main findings | Main symptoms and/or important clinical findings. | Abstract, Results. |
| 3c | Abstract – Diagnoses/interventions/outcomes | Main diagnoses, therapeutic interventions, and outcomes. | Abstract, Methods and Results. |
| 3d | Abstract – Conclusion | Main "take-away" lesson(s). | Abstract, Conclusions. |
| 4 | Introduction | Brief background summary referencing relevant literature. | Introduction (3 paragraphs; Chari 2022, Laheij 2023, Pandeshwar 2015, Grossmann 2025). |
| 5a | Patient information | De-identified patient-specific information. | Table 1: age/sex, myeloma status, prior lines of therapy, for all four patients. |
| 5b | Patient information | Primary concerns and symptoms. | Results, paragraphs 2–3; Table 1 (onset, grade, duration of dysgeusia and xerostomia). |
| 5c | Patient information | Medical, family, and psychosocial history, including relevant genetic information. | Partially reported: myeloma status and prior treatment lines (Table 1). Detailed family/psychosocial/genetic history is outside the scope of this oral-toxicity–focused brief report and was not systematically collected; noted implicitly by the report's focus. |
| 5d | Patient information | Relevant past interventions and their outcomes. | Prior lines of myeloma therapy summarized categorically in Table 1 (e.g., "fifth-line treatment"); individual regimen detail not reported, consistent with the brief-report format. |
| 6 | Clinical findings | Relevant physical examination and other significant clinical findings. | Table 1, "Baseline oral examination findings" row (all four patients); Results, paragraphs 2–3 (absence of mucositis/oral pain; reduced salivary film). |
| 7 | Timeline | Historical and current information from this episode of care, organized as a timeline (table or figure). | Table 1 provides a per-patient timeline (onset day, PBM start day, number of sessions, symptom duration, follow-up duration) for all four patients. A dedicated visual timeline figure was not created but can be added if requested by the editor. |
| 8a | Diagnostic assessment | Diagnostic methods (PE, laboratory, imaging, surveys). | Methods, "Clinical evaluation": structured interview and oral examination; no sialometry or psychophysical gustatory testing performed (stated as a limitation). |
| 8b | Diagnostic assessment | Diagnostic challenges. | Discussion, paragraph 1 (new sentence): addresses the differential from primary burning mouth syndrome, based on temporal association with talquetamab and normal baseline oral findings. |
| 8c | Diagnostic assessment | Diagnostic reasoning, including other diagnoses considered. | Discussion, paragraph 1, same sentence as 8b; Methods, "Eligibility criteria" (exclusion of pre-existing taste alteration and other confounding conditions at baseline). |
| 8d | Diagnostic assessment | Prognostic characteristics where applicable. | Not applicable to myeloma prognosis; oral-toxicity course (resolution/persistence) reported per patient in Table 1. |
| 9a | Therapeutic intervention | Types of intervention (pharmacologic, surgical, preventive, self-care). | Methods, "Photobiomodulation protocol" and "Clinical evaluation"; Results (concomitant xylitol mouthwash and saliva substitutes). |
| 9b | Therapeutic intervention | Administration of intervention (dosage, strength, duration). | Methods and Table 2 (complete PBM dosimetry); Table 1 (talquetamab dose/schedule and PBM session counts per patient). |
| 9c | Therapeutic intervention | Changes in intervention with explanation. | Discussion, paragraph 5 (sessions concentrated early, then spaced as symptoms stabilized); Table 1, "Treatment interruptions" row. |
| 10a | Follow-up and outcomes | Clinician- and patient-assessed outcomes. | Table 1 (CTCAE grade/duration for dysgeusia and xerostomia, weight change, follow-up status) for all four patients. |
| 10b | Follow-up and outcomes | Important follow-up diagnostic and other test results. | Not performed (no quantitative salivary flow or gustatory testing); stated as a limitation in Discussion. |
| 10c | Follow-up and outcomes | Intervention adherence and tolerability, and how this was assessed. | Methods, "Safety assessment"; Results (adherence to xylitol/saliva substitutes was self-reported, not independently verified; no PBM-related adverse events). |
| 10d | Follow-up and outcomes | Adverse and unanticipated events. | Methods, "Safety assessment"; Results, final paragraph (no PBM-related adverse events in any patient). |
| 11a | Discussion | Strengths and limitations in the approach to this case/series. | Discussion, Limitations paragraph. |
| 11b | Discussion | Discussion of relevant medical literature. | Discussion, paragraphs 2–4 (Fleischer 2025, Laheij 2023, Kayahara 2025, Ferreira 2024, Grossmann 2025). |
| 11c | Discussion | Scientific rationale for any conclusions. | Discussion, paragraph 3 (mechanistic rationale, explicitly framed as hypothesis, not demonstrated finding). |
| 11d | Discussion | Primary "take-away" lessons, without overgeneralizing. | Conclusion (feasibility and tolerability only; efficacy not established). |
| 12 | Patient perspective | Patient's perspective or experience, where appropriate. | Not collected. Patient-reported outcome/experience data beyond structured symptom interviews were not part of this protocol; this is noted as a limitation and an opportunity for future prospective studies. |
| 13 | Informed consent | Did the patient give informed consent? Provide if requested by the journal. | Declarations, "Consent to publish": written informed consent for publication of clinical data obtained from all four patients; ethics approval CAAE 96537226.8.0000.0071. |

*Items 5c, 7, and 12 are only partially addressed or not applicable, consistent with the scope and format of a brief report of a four-patient descriptive series; this is stated transparently in the checklist above rather than overstated in the manuscript.*
